# Novel Mutation Glu98Lys in Cardiac Tropomyosin Alters Its Structure and Impairs Myocardial Relaxation

**DOI:** 10.3390/ijms241512359

**Published:** 2023-08-02

**Authors:** Alexander M. Matyushenko, Victoria V. Nefedova, Anastasia M. Kochurova, Galina V. Kopylova, Natalia A. Koubassova, Anna G. Shestak, Daria S. Yampolskaya, Daniil V. Shchepkin, Sergey Y. Kleymenov, Natalia S. Ryabkova, Ivan A. Katrukha, Sergey Y. Bershitsky, Elena V. Zaklyazminskaya, Andrey K. Tsaturyan, Dmitrii I. Levitsky

**Affiliations:** 1 A.N. Bach Institute of Biochemistry, Research Center of Biotechnology, Russian Academy of Sciences, Moscow 119071, Russia; ammatyushenko@mail.ru (A.M.M.); victoria.v.nefedova@mail.ru (V.V.N.); daria_logvinova@mail.ru (D.S.Y.); s.yu.kleymenov@gmail.com (S.Y.K.); 2 Institute of Immunology and Physiology, Ural Branch of Russian Academy of Sciences, Yekaterinburg 620049, Russia; kochurova.a.m@mail.ru (A.M.K.); g_rodionova@mail.ru (G.V.K.); cmybp@mail.ru (D.V.S.); serg.bersh@gmail.com (S.Y.B.); 3 Institute of Mechanics, Moscow State University, Moscow 119192, Russia; nkoubassova@gmail.com (N.A.K.); andrey.tsaturyan@gmail.com (A.K.T.); 4 Petrovsky National Research Centre of Surgery, Moscow 119991, Russia; anna.shestak87@gmail.com (A.G.S.); helenezak@gmail.com (E.V.Z.); 5 Koltzov Institute of Developmental Biology, Russian Academy of Sciences, Moscow 119334, Russia; 6 Department of Biochemistry, Faculty of Biology, Lomonosov Moscow State University, Moscow 119234, Russia; n.ryabcova@gmail.com (N.S.R.); katrukhai@mail.ru (I.A.K.); 7 HyTest Ltd., 20520 Turku, Finland; 8 N.P. Bochkov Research Centre for Medical Genetics, Moscow 20520, Russia

**Keywords:** tropomyosin, cardiomyopathy-causing mutations, complex cardiomyopathy, circular dichroism, differential scanning calorimetry, in vitro motility assay, molecular dynamics

## Abstract

We characterized a novel genetic variant c.292G > A (p.E98K) in the *TPM1* gene encoding cardiac tropomyosin 1.1 isoform (Tpm1.1), found in a proband with a phenotype of complex cardiomyopathy with conduction dysfunction and slow progressive neuromuscular involvement. To understand the molecular mechanism by which this mutation impairs cardiac function, we produced recombinant Tpm1.1 carrying an E98K substitution and studied how this substitution affects the structure of the Tpm1.1 molecule and its functional properties. The results showed that the E98K substitution in the N-terminal part of the Tpm molecule significantly destabilizes the C-terminal part of Tpm, thus indicating a long-distance destabilizing effect of the substitution on the Tpm coiled-coil structure. The E98K substitution did not noticeably affect Tpm’s affinity for F-actin but significantly impaired Tpm’s regulatory properties. It increased the Ca^2+^ sensitivity of the sliding velocity of regulated thin filaments over cardiac myosin in an in vitro motility assay and caused an incomplete block of the thin filament sliding at low Ca^2+^ concentrations. The incomplete motility block in the absence of Ca^2+^ can be explained by the loosening of the Tpm interaction with troponin I (TnI), thus increasing Tpm mobility on the surface of an actin filament that partially unlocks the myosin binding sites. This hypothesis is supported by the molecular dynamics (MD) simulation that showed that the E98 Tpm residue is involved in hydrogen bonding with the C-terminal part of TnI. Thus, the results allowed us to explain the mechanism by which the E98K Tpm mutation impairs sarcomeric function and myocardial relaxation.

## 1. Introduction

Inherited cardiomyopathies are known to be caused by many missense mutations in different genes, including those encoding various muscle proteins. Among them, hypertrophic cardiomyopathy (HCM) is the most common inherited heart disease, caused by mutations in more than 20 genes [1], including the *TPM1* gene encoding cardiac tropomyosin (Tpm, Tpm1.1, or α-Tpm isoform) [2,3]. Sarcomere hypercontractility leads to the progressive thickening of the left ventricular wall and the incomplete relaxation that underlies diastolic dysfunction. Both factors are the key components in producing the clinical phenotype of HCM [4]. The danger of these changes is confirmed by the fact that left-ventricle hypertrophy is the most common cause of sudden death in young adults [5].

About 5% of all known HCM cases are associated with mutations in the *TPM1* gene encoding the cardiac Tpm1.1 isoform [4,6,7,8]. This Tpm isoform plays, together with the troponin (Tn) complex, a key role in the Ca^2+^ regulation of contraction of cardiac and fast skeletal muscles [9,10]. Tpm is a genuine actin-binding protein that forms a ropelike structure along the entire length of the actin filament due to the end-to-end polymerization of the Tpm molecules. In the absence of Ca^2+^, it sterically blocks the myosin-binding sites on actin. Ca^2+^ binding to Tn during muscle activation leads Tpm to move away from the blocking position and allows the binding of myosin heads to actin.

In terms of structure, the Tpm molecule is a typical α-helical coiled-coil dimer whose amino acid sequence contains a heptad repeat (*a-b-c-d-e-f-g*)*_n_* in which residues at positions *a* and *d* are hydrophobic and form a hydrophobic core of the molecule while polar residues at positions *e* and *g*, typically of opposite charge, form electrostatic interchain interactions and additionally stabilize the coiled-coil structure [10]. HCM-associated mutations often occur at the *g* and *e* positions of the hepta-peptide repeat [4,6], and some of them, such as Asp175Asn, Glu180Gly, and Glu180Val, can significantly affect the structural and functional properties of Tpm1.1 [11,12].

About 20 mutations associated with HCM have been identified in the *TPM1* gene [4,7,8]. For understanding the mechanism by which these mutations lead to HCM development, it is important to know how they affect the structural and functional properties of Tpm1.1. It has been shown that most previously studied mutations affect the structural and regulatory properties of Tpm1.1 and its interaction with actin and/or the Tn complex [8,11,12,13,14,15,16,17]. Among them, the effects of the D175N and E180G mutations are the most intensively characterized. However, this information is absent for many other HCM-associated mutations in the *TPM1* gene, which were found in genetic studies or during clinical testing.

In the present work, we identified genetic variant c.292G > A (p.E98K) in the TPM1 gene that was recently registered in the ClinVar database (first submission 21 October 2021). This variant had risen de novo and was assigned to HCM without any clinical description (National Center for Biotechnology Information. ClinVar; (VCV001472862.2) https://www.ncbi.nlm.nih.gov/clinvar/variation/VCV001472862.2 (Last update 7 February 2023)). However, this mutation in the *g* position was assessed as likely pathogenic without any data about its effect on the structural or functional properties of Tpm.

We produced recombinant E98K Tpm1.1 and studied its properties using various biochemical and biophysical methods. We also performed a molecular dynamics (MD) simulation to understand how the E98K mutation affects the Tpm1.1 coiled-coil structure and Tpm’s ability to regulate actin–myosin interactions.

The results showed that the E98K substitution significantly alters both the structure of the Tpm1.1 molecule and its functional and regulatory properties, including the interaction between Tpm and Tn, although it has no significant effect on Tpm’s affinity for actin. The results of the MD simulation revealed possible molecular mechanisms underlying the changes found in vitro and observed clinically.

## 2. Results

### 2.1. Genetic Evaluation and Detection of E98K Mutation

We performed clinical and instrumental investigations (Resting and 24-h Holter Monitoring ECG, EchoCG), genetic counseling, and DNA testing for a 44-year-old (y.o.) male proband with a complex cardiac and neuromuscular phenotype. The follow-up time was 6 years (proband is 50 y.o. now).

**Figure 1:**
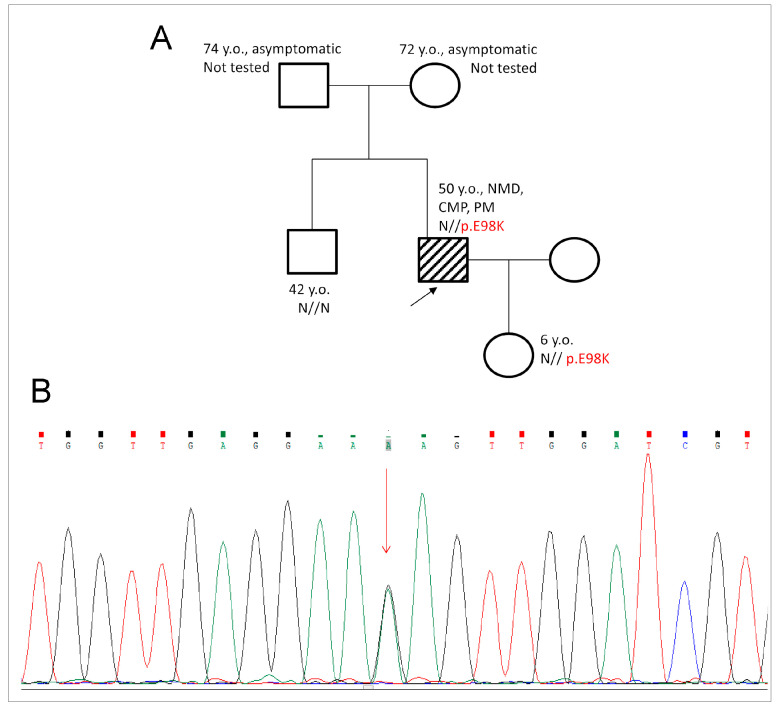
(**A**). Pedigree of the family T. The affected proband is shown with the closed symbol and is marked by the black arrow. The age of family members is shown at the last appointment (early 2023). CMP—cardiomyopathy, NMD—neuromuscular disease, PM—pacemaker. (**B**). Fragment of the Sanger electropherogram of the *TPM1* gene. Visualization is given by the Chromas2.5 software (Technelysium Pty. Ltd., South Brisbane, Australia). (A—adenine, green peaks; C—cytosine, blue peaks; G—guanine, black peaks; T—thymine, red peaks). Heterozygous variant c.292G > A (p.E98K) is marked by the red arrow.

Anamnesis: The family history was unremarkable (Figure 1A). The first clinical symptoms appeared at the age of 38–39 and included pre-syncope, low tolerance for exercise, mild weakness of the distal skeletal muscles, loss of muscle mass and volume, and palpitations. Further investigation revealed rhythm and conduction abnormalities such as prominent bradyarrhythmia, rhythm pauses of more than 3 s, and atrial fibrillation, which required a dual-chamber pacemaker implantation at the age of 40. Cardiomyopathy was characterized as HCM with diastolic dysfunction. From a young age, the patient could not run.

The patient was 44 y.o. at the time of the first genetic consultation and subsequent DNA testing. Cardiac remodeling was complex and included features characteristic of several cardiomyopathies. Mild cardiac hypertrophy in the mid-ventricular level up to 15 mm without left-ventricular outflow-tract obstruction (LVOT) was diagnostic for HCM. A prominent restrictive left-ventricular filling pattern with bilateral atriomegaly was characteristic of restrictive cardiomyopathy (RCM). The diffuse reduction of cardiac contractility with an ejection fraction (LV EF) of 48–50% can be interpreted as a manifestation of cardiac dilation (DCM). We found a rare heterozygous SNV variant at chr15:63349235 (hg19) c.292G > A (p.Glu98Lys, p.E98K) in the *TPM1* gene. The presence of this variant was tested by capillary Sanger sequencing using Applied Biosystem 3500 Genetic Analyzer (ThermoFisher Scientific, Waltham, MA, USA) in the proband and his clinically unaffected daughter (6 y.o., Figure 1B). Other relatives (brother and parents) were healthy and refused genetic testing. The pathogenicity of this variant was assessed in accordance with ACMG (2015) criteria [18] as likely pathogenic (Class IV).

It should be noted that this variant was found only in the *TPM1* gene. No additional pathogenic, likely pathogenic variant, nor unique variant of unknown clinical significance was found in the scope of the genetic testing of the other 25 sarcomeric and non-sarcomeric genes of the patient.

To evaluate the pathogenic potential of this mutation in the *TPM1* gene, we produced recombinant E98K and WT Tpm proteins, which have an Ala-Ser N-terminal extension to imitate the naturally occurring N-terminal acetylation of Tpm. We applied various methods to investigate how the E98K substitution affects the structural and functional properties of Tpm.

### 2.2. Effects of the E98K Mutation on the Structure of Tpm Molecule

#### 2.2.1. Thermal Unfolding of E98K Tpm Mutant Studied with Circular Dichroism (CD) and Differential Scanning Calorimetry (DSC)

**Figure 2:**
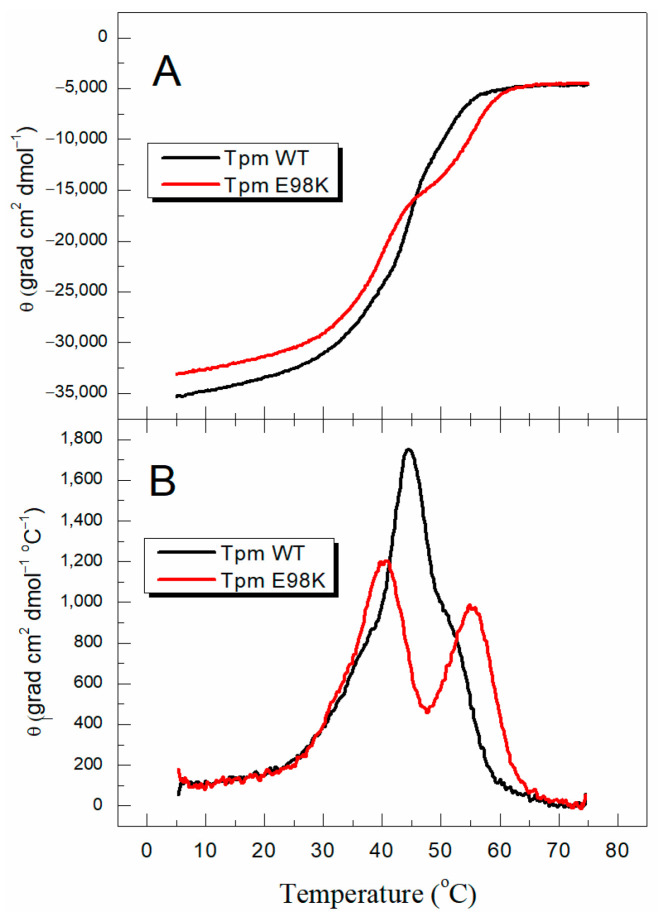
CD measurements of the thermal unfolding of E98K Tpm compared with that of WT Tpm. (**A**) The temperature dependencies of α-helix content were measured as the ellipticity at 222 nm at a constant heating rate of 1 °C/min. (**B**) First-derivative profiles for the data shown in panel (**A**).

At first, we applied CD to study the effects of the E98K mutation on the Tpm secondary structure. The CD spectrum recorded at 5 °C for the E98K Tpm mutant was typical of wild-type (WT) Tpm with two negative maxima at 208 and 222 nm, characteristic of an α-helical coiled-coil protein (see Supplementary Figure S1). The thermal stability of E98K Tpm compared to WT Tpm was examined using CD by measuring the ellipticity at 222 nm (Figure 2A). The results showed that the E98K mutation caused a decrease in Tpm thermal stability at all temperatures below 45 °C, while it stabilized the Tpm molecule within the range from 45 °C to 60 °C. The stabilizing and destabilizing effects of the E98K mutation can be observed more clearly when the curves are plotted in their differential form, as the first-order derivative (Figure 2B). There are two major transitions of cooperative thermal unfolding for E98K Tpm, with maxima at 40.3 °C and 55 °C. Comparing these data with those for WT Tpm (Figure 2B), we conclude that the E98K mutation strongly destabilizes a significant part of the Tpm molecule and slightly stabilizes another part.

**Figure 3:**
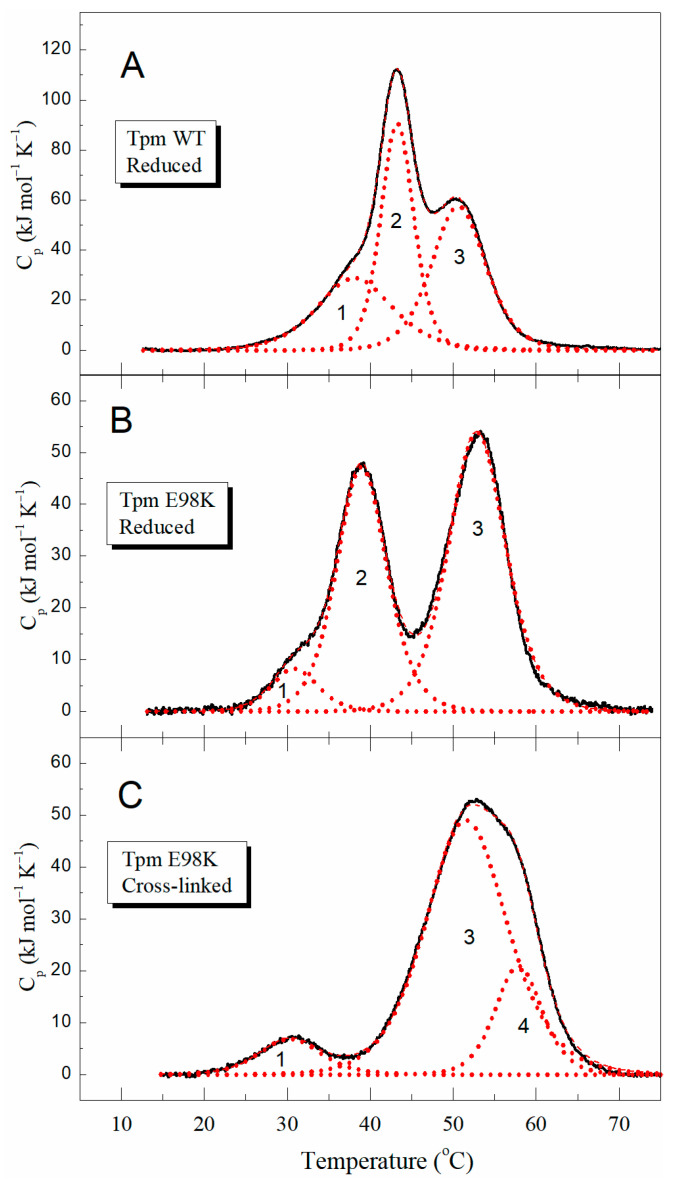
Temperature dependences of the excess heat capacity (*C_p_*) monitored by DSC and deconvolution analysis of the heat sorption curves for WT Tpm in the fully reduced state (**A**) and Tpm with mutation E98K (**B**,**C**) both in reduced (**B**) and in cross-linked (**C**) states. Solid lines represent the experimental curves after the subtraction of instrumental and chemical baselines, and dotted red lines represent the individual thermal transitions (calorimetric domains 1–4) obtained from fitting the non-two-state model [19] to the data.

To study the thermal unfolding and domain structure of the E98K Tpm mutant in detail, we applied DSC, the method allowing for the deconvolution of the thermal unfolding curve into individual thermal transitions (calorimetric domains) in the Tpm molecule. The heat-induced unfolding of WT Tpm and E98K Tpm species was fully reversible, thus making possible further deconvolution analysis of their DSC curves. Figure 3A,B show DSC profiles for E98K Tpm in its entirely reduced state, compared with that for reduced WT Tpm, and the results of their deconvolution into individual thermal transitions corresponding to separate calorimetric domains in the molecule. The main calorimetric parameters for these domains (*T_m_* and ΔH_cal_) are summarized in Table 1.

Both WT Tpm and E98K Tpm in the reduced state demonstrated three calorimetric domains on their DSC curves (Figure 3A,B). Two of them, domains 2 and 3, were assigned in previous DSC studies of WT Tpm to the thermal unfolding of the C- and N-terminal parts of the Tpm molecule, respectively [11,20]; as for the least thermostable domain 1, it was supposed to correspond to the melting of some other parts of the molecule, such as its middle part or the head-to-tail overlap junction between the N- and C-termini of neighbor Tpm molecules [20,21]. The E98K mutation caused significant changes in the thermal unfolding of the Tpm molecule: it strongly destabilized calorimetric domain 2 by shifting its thermal transition by more than 4 °C to a lower temperature and stabilized calorimetric domain 3 by increasing its transition temperature by 2.2 °C (Figure 3B, Table 1).

It is important to note that destabilized domain 2 of E98K Tpm coincides in position with the least thermostable domain 1 of WT Tpm (Figure 3A,B; Table 1). One can suppose from these data that these domains in E98K Tpm, a significant part of domain 1 and destabilized domain 2, either melt together as domain 2 or their thermal transitions coincide in position and cannot be separated by deconvolution analysis. As a result, only some small part of domain 1 can be observed on the DSC thermogram of E98K Tpm as a separate thermal transition (domain 1 in Figure 3B, which melts at a very low temperature (30.9 °C) and with very low enthalpy (Table 1)). It is not excluded that some other parts of the E98K Tpm molecule melt non-cooperatively at very low temperatures (see Figure 2A), and this may explain, at least partly, why the total enthalpy of the thermal unfolding is less for this Tpm mutant than for WT Tpm (Table 1).

Thus, the DSC results presented in Figure 3A,B show that the thermal unfolding of E98K Tpm is quite different from that of WT Tpm. This observation prompted us to carefully identify the calorimetric domains in the E98K Tpm molecule, i.e., to reveal their correspondence to certain parts of the molecule. For this purpose, we performed a cross-linking of E98K Tpm by the formation of a disulfide bond between Cys residues in two chains of the E98K Tpm dimer. The cross-linking was previously shown to significantly increase the thermal stability of the Tpm region where Cys residues are located [11,20]. The E98K Tpm, like WT Tpm, contains the only Cys residue, Cys190, in the C-terminal half of each chain of the Tpm dimer. Previous studies with WT Tpm have shown that the cross-linking of these residues significantly increases the thermal stability of calorimetric domain 2, which reflects the melting of the C-terminal part of the Tpm molecule [11,20]. In the present work, we performed similar DSC experiments with the E98K Tpm mutant. The cross-linking of E98K Tpm was achieved by several subsequent heatings of the sample directly in the measuring cell of the calorimeter in the absence of reducing agents, as described earlier [22]. The entirely cross-linked state of the E98K Tpm was achieved after the fifth heating of the sample (see Figure 3C).

Comparing the DSC data obtained for the cross-linked E98K Tpm (Figure 3C) with those for the reduced E98K Tpm (Figure 3B), one can observe that the cross-linking causes a complete disappearance of calorimetric domain 2 from the thermogram with no effect on the least thermostable domain 1. On the other hand, a new domain 4 with a maximum of 57.9 °C appears on the thermogram, while the calorimetric enthalpy of former domain 3 strongly increases (Figure 3C; Table 1). One can propose from these data that a significant part of former domain 2, being stabilized by the cross-linking, melts together with domain 3. However, it seems more likely that these two domains, domain 2 stabilized by the cross-linking and domain 3, whose thermal transitions overlap on the thermogram, simply cannot be correctly separated upon deconvolution analysis. In any case, the results of cross-linking experiments indicate that calorimetric domain 2 (Figure 3B), whose stability strongly increased due to disulfide cross-linking upon heating (domain 4 and some part of domain 3 in Figure 3C), reflects the thermal unfolding of the C-terminal part of the E98K Tpm molecule where Cys190 is located.

Taking together all the DSC data presented in Figure 3, we can conclude that the E98K substitution strongly destabilizes the C-terminal part of the Tpm molecule.

#### 2.2.2. Solution Viscosity of E98K Tpm Mutant

The effect of the E98K Tpm mutation on the interaction of the ends of the Tpm molecules was estimated by viscosity measurement. The viscosity of E98K Tpm solution, after subtraction of the buffer viscosity (see Section 4), did not significantly differ from that for WT Tpm: 0.28 ± 0.01 mPa∙s for E98K Tpm vs. 0.31 ± 0.01 mPa∙s for WT Tpm (mean ± SEM). This indicates that the E98K mutation does not affect the interaction between the N- and C-ends of adjacent Tpm molecules.

#### 2.2.3. Molecular Dynamics (MD) Simulation

To compare the structural characteristics of E98K Tpm with those of WT Tpm in silico, we performed the MD simulation of Tpm dimers in the presence of an explicit solvent (TIP3P water, Na^+^ and Cl^−^ ions) using the GROMACS MD software with the AMBER99SB-ILDN or CHARMM36 force field and periodic boundary conditions as described in Methods (see Section 4 for details). The stability of the α-helical structure of the Tpm dimers was estimated from the average occupancy of the backbone hydrogen bonds (h-bonds) within the two chains of the Tpm coiled coil. MD trajectories that were 204.8 ns long were calculated, and their snapshots were recorded every 200 ps for further analysis.

**Figure 4:**
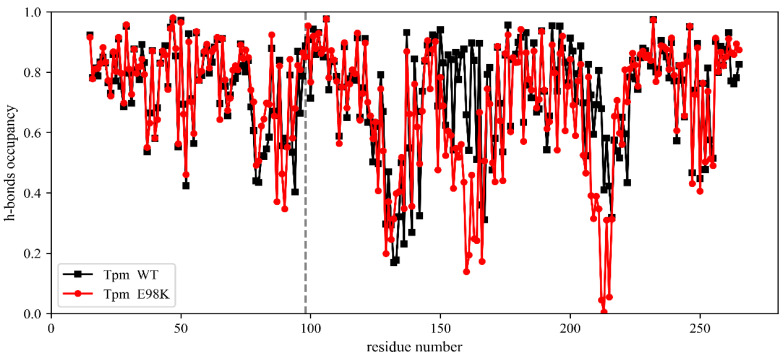
The time-average occupancies of the backbone h-bonds for WT Tpm (black) and E98K Tpm (red). The average occupancy values in the two chains of the Tpm coiled coil are shown against the residue number. The vertical dashed line shows the position of the 98th residue.

The time-average occupancies of the hydrogen bonds (h-bonds) within the backbones of two Tpm α-helical chains for WT Tpm and E98K Tpm are shown in Figure 4. The time-averaging was performed only for the second half of the MD trajectory when the process became quasi-steady.

No significant changes in the h-bond occupancies were found in the vicinity of the 98th Tpm residue (Figure 4). The α-helical structure of the whole N-terminal part of Tpm was very stable for both WT Tpm and E98K Tpm. In contrast, the decrease in the occupancy of the α-helical h-bonds upon the E98K substitution was observed in the central part of the molecule, within the region of Tpm residues 160–165 and, especially, in the vicinity of the non-canonical 218th residue. This simulation result suggests that the E98K substitution may have a long-range destabilizing effect on the coiled-coil structure of the central and C-terminal parts of the Tpm molecule.

### 2.3. Effects of the E98K Substitution on Tpm Functional Properties

#### 2.3.1. Affinity of E98K Tpm Mutant to F-Actin

**Figure 5:**
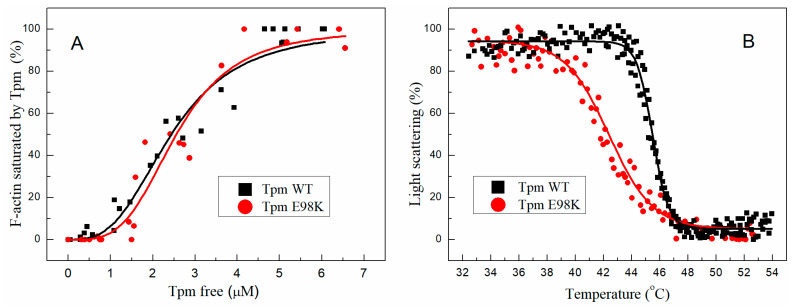
The effect of the E98K substitution in the Tpm molecule on the Tpm affinity for F-actin estimated by the co-sedimentation assay (**A**) and on the thermal stability of the Tpm-actin complex determined by light scattering measurements (**B**).

The E98K substitution did not significantly affect the Tpm affinity to F-actin assessed using a co-sedimentation assay (Figure 5A and Supplementary Figure S2). The *K*_50%_ values, corresponding to the Tpm concentration at which half of the actin becomes saturated, were 2.57 ± 0.12 µM for E98K Tpm vs. 2.45 ± 0.40 µM for WT Tpm.

#### 2.3.2. Effect of E98K Substitution on the Thermal Stability of the F-Actin–Tpm Complex

The thermal stability of the F-actin–Tpm complex was estimated with light scattering whose thermally induced decrease reflects the dissociation of the complex (Figure 5B). The E98K substitution in the Tpm molecule significantly destabilized the Tpm complex with F-actin. The *T_diss_* values (i.e., the temperature at which a 50% decrease in the light scattering occurs) were equal to 42.4 ± 0.02 °C for E98K Tpm vs. 45.5 ± 0.03 °C for WT Tpm. These results correlate with the DSC data showing that the E98K substitution causes a significant decrease in the thermal stability of the C-terminal half of the Tpm molecule (calorimetric domain 2 in Figure 3 and Table 1). This agrees well with the results of previous studies [12,21,23] indicating that the stability of the Tpm–F-actin complexes depends rather on the thermal stability of the Tpm molecule than on Tpm’s affinity to actin.

#### 2.3.3. Effect of E98K Substitution on Tpm Regulatory Properties

**Figure 6:**
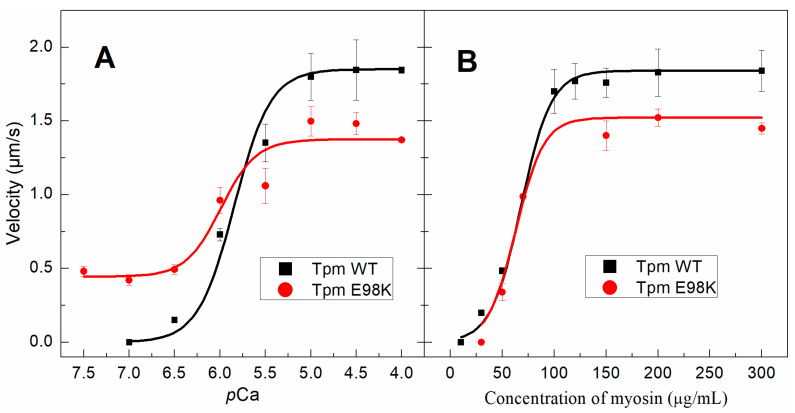
Dependence of the sliding velocity of reconstructed thin filaments containing WT Tpm or E98K Tpm over cardiac myosin in the in vitro motility assay on *p*Ca at myosin concentrations of 300 µg/mL (**A**) on myosin concentration at saturating calcium concentration (*p*Ca 4) (**B**). Each data point represents the mean ± SD from three experiments. The data were fitted with the Hill equation. Characteristics of the *p*Ca–velocity relationship are presented in Table 2.

We investigated the effects of the E98K Tpm substitution on the Ca^2+^-regulation of actin–myosin interactions by analyzing the Ca^2+^ dependence of the sliding velocity of thin filaments, reconstructed from F-actin, Tpm, and Tn over cardiac ventricular myosin in an in vitro motility assay (Figure 6A, Table 2). The substitution led to a decrease in the maximum sliding velocity (*V*_max_) at saturating Ca^2+^ concentrations, a significant increase in the velocity at a low Ca^2+^ concentration (*p*Ca ≥ 6.5), and elevation of Ca^2+^ sensitivity of the sliding rate expressed as *p*Ca_50_ (*p*Ca value at which the velocity is half-maximal, Table 2). The increased Ca^2+^ sensitivity and incomplete relaxation at low Ca^2+^ concentrations are known as the characteristic features of HCM [4,7,8,24,25].

The number of moving thin filaments was measured as described earlier [12] at the saturated Ca^2+^ concentration (at *p*Ca 4) and at *p*Ca 7.0–7.5. At *p*Ca 7.0, all WT Tpm-containing filaments stuck, while ~50% of the filaments with the E98K Tpm mutant still moved even at *p*Ca 7.5. At the saturated Ca^2+^ concentration, all filaments with any of these two Tpms moved smoothly.

We also measured the dependence of the sliding velocity of the regulated thin filaments on the myosin concentration at the saturating (*p*Ca 4) calcium concentration (Figure 6B) to study the effect of the E98K Tpm substitution on the cross-bridge−cross-bridge cooperativity of actin–myosin interactions [26]. The higher the cooperativity, the fewer myosin heads are needed to move the thin filament at the half-maximal velocity. The myosin concentration required to achieve the half-maximal velocity (*C*_50_) of thin filaments containing E98K (63.0 ± 1.0 µg/mL) did not differ from that of the filaments with WT Tpm (67.9 ± 2.0 µg/mL). Thus, the E98K Tpm substitution did not affect the cooperativity of myosin interactions with thin filaments at full activation.

#### 2.3.4. Possible Effect of E98K Substitution in Tpm on Its Interaction with TnI in the F-Actin–Tpm–Tn Complex (MD Simulation)

**Figure 7:**
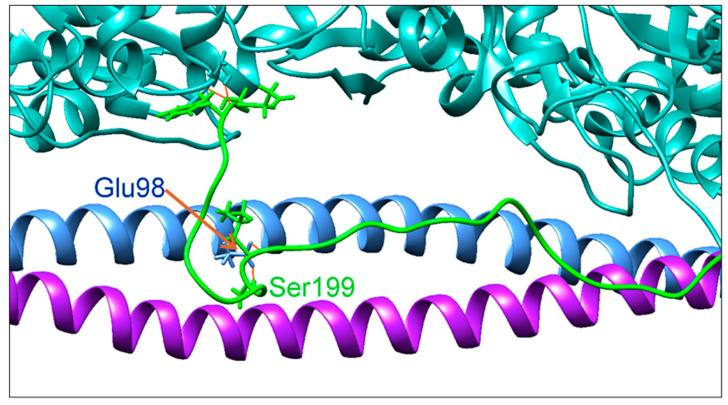
A snapshot of the MD simulation trajectory. Actin monomers (sea green), WT Tpm (cornflower blue and purple), and Tn (Tn-I C-terminal fragment, green) are shown. During the simulation, Tpm Glu98 formed the h-bonds with Ser199 and Leu198 of TnI, while C-terminal residues 208–210 of TnI were anchored in the actin monomer. H-bonds are shown in red.

The in vitro motility assay data obtained with the regulated thin filament suggest that the E98K Tpm substitution impairs muscle relaxation and facilitates the transition of the thin filament from the blocked to the “open” state (Figure 6A, Table 2). To understand the possible molecular mechanism of these changes, we performed an MD simulation of the F-actin–Tpm–Tn complex, using a recently published refined atomic model of a segment of the thin filament of cardiac muscle [27] based on high-resolution cryo-EM data [28,29]. The results of this simulation revealed the possible involvement of the E98 Tpm residue in the interaction between Tpm and TnI in the blocked state of the regulatory unit (Figure 7).

The results of the MD simulation of the F-actin–Tpm–Tn complex showed that the Glu98 Tpm residue interacts with a flexible C-terminal loop of TnI. The C-terminus of TnI is anchored in an actin monomer, keeping the regulatory complex in the blocked state while h-bonds between the side chain of Glu98 and TnI residues hold Tpm and TnI together. The substitution of negatively charged Glu98 Tpm residue for positively charged Lys may loosen the Tpm–TnI interaction and increase Tpm motility that, in its turn, may partially unlock myosin binding site(s) on nearby actin monomer(s). This observation may explain the incomplete block of actin–myosin motility in the absence of Ca^2+^ in vitro upon the Glu98Lys Tpm substitution.

## 3. Discussion

We investigated the structural and functional properties of Tpm with the E98K substitution. Two major changes caused by this mutation were found: a long-distance destabilization of the coiled-coil structure of the Tpm molecule and an impairment of the blocked state of the thin filament.

### 3.1. Long-Distance Destabilization of Tpm Molecule upon Glu98Lys Substitution

The Glu98 Tpm residue is located in the N-terminal part of the molecule at the *g* position in the heptad repeat (see Supplementary Figure S3). Its substitution for Lys increased the thermal stability of calorimetric domain 3 (Figure 3, Table 1), associated with the thermal unfolding of this part of Tpm molecule [11,20]. On the other hand, this substitution led to a significant thermal destabilization of calorimetric domain 2, associated with the unfolding of the C-terminal part of the molecule [11,20], located far from the 98th residue in the coiled-coil Tpm molecule. The DSC data (Figure 3, Table 1) suggesting a long-distance destabilizing effect of this substitution were supported by the results of the MD simulation of Tpm that predict a decrease in the occupancies of the backbone hydrogen bonds in the central and C-terminal parts of both Tpm α-helices (Figure 4). We do not have a specific explanation of the long-distance destabilization effect of the Glu98Lys Tpm substitution, although the possibility of the long-range effects of Tpm substitutions on its stability was observed previously [15,20,23,30]. For example, it was shown using DSC that Glu218Leu substitution in the C-terminal part of the Tpm molecule strongly increases the thermal stability of the N-terminal part of the molecule [23]; Ly and Lehrer showed that an HCM-associated Glu180Gly mutation formed a new site for Tpm cleavage with trypsin at the K233 residue, located at a substantial distance from the mutation site [15]. In any case, these results demonstrate the high cooperativity of the Tpm coiled-coil structure, where a single substitution in the primary sequence can lead to global changes in other parts of the Tpm molecule located at a significant distance from the point of substitution.

### 3.2. Glu98Lys Tpm Substitution Destabilizes the Blocked State of the Thin Filaments and Probably Causes HCM with Restrictive Phenotype

The effect of the Glu98Lys substitution on the Tpm regulatory properties studied with the in vitro motility assay showed that this substitution leads to an incomplete block of the thin filament sliding at low Ca^2+^ concentration and an increase in the Ca^2+^ sensitivity of the filament velocity (Figure 6A, Table 2). At a *p*Ca of 7.5, the sliding velocity of reconstructed thin filaments containing Glu98Lys Tpm was 32% of its maximal value at *p*Ca 4. The results of the MD simulation of the F-actin–Tpm–Tn complex in the blocked state suggest a possible molecular mechanism of the incomplete relaxation of the thin filaments with the Gly98Lys Tpm in the absence of Ca^2+^. The C-terminal of TnI anchors the Tpm–Tn strand on actin and keeps the regulatory proteins in the blocked state [28,29]. Our MD simulation suggests that the Tpm residue Glu98 participates in the TnI–Tpm interaction, forming hydrogen bonds with the side chain of TnI residue Ser199 and a backbone atom of neighbor residue Leu198 (Figure 7). The position of Tpm residue Glu98 near TnI Ser199 and its possible involvement in the Tpm–TnI interaction were pointed out by Lehman et al. [31,32]. Substituting negatively charged Glu98 for positively charged Lys would break the h-bonds between Tpm and the C-terminal part of TnI and, therefore, loosen the Tpm–TnI interaction. The Tpm–TnI decoupling would increase Tpm mobility on the surface of the actin filament, leading to a partial unlocking of the myosin binding sites on actin monomer(s) near the Tpm 98 residue. As a result, myosin heads could bind actin next to Tpm residue 98 even at low Ca^2+^ concentration, thus leading to an incomplete blocking of the actin–myosin interaction.

The in vitro motility assay data reveal the possible molecular mechanism of the diastolic dysfunction found in the patient with the Glu98Lys substitution. According to Lehrer and Geeves [33], the HCM with the symptoms of restrictive refilling of the left ventricle is often linked with the incomplete inhibition of the actin–myosin interaction at low Ca^2+^ levels. Presumably, in our case, it was caused by the disturbance of the Tpm–TnI interaction.

The restrictive phenotype is a rare variant of HCM presentation accounting for about 2.5% of affected families and is generally associated with a poor prognosis; mutations in the *MYH7* and *TNNI3* genes encoding cardiac myosin II and TnI, respectively, were found previously in probands with complex cardiomyopathies with mild hypertrophy and severe restriction [34]. The genetic variant studied in this work was previously revealed in another population, submitted in the ClinVar database on 21 October 2021, and assessed as likely pathogenic without providing data about its effect on the properties of Tpm and its interaction with its partner proteins. In the present work, we provided clinical data and the results of various in vitro studies, which demonstrate that the Glu98Lys Tpm substitution can cause an incomplete block of the actin–myosin interaction at low Ca^2+^ concentrations, impairing myocardial relaxation and leading to diastolic dysfunction and HCM with restrictive phenotype.

### 3.3. Other HCM-Associated Mutations in the N-Terminal Part of the Tpm Molecule

Several cardiomyopathy-associated mutations in pseudo-repeat 3 of Tpm near Tpm residue 98 have been described [35]. Among them, the Val95Ala substitution has been studied in the most detail [36,37,38]. It causes HCM with a mild phenotype but poor prognosis [36], slightly decreases Tpm’s affinity for F-actin in the absence of Tn, and slightly increases the Ca^2+^ sensitivity of myosin S1 ATPase in the presence of regulated thin filaments [37] and their sliding velocity over a myosin-covered surface in vitro [38]. These changes can result from a destabilization of the Tpm coiled coil caused by a substitution of canonical hydrophobic residue Val95 in the *d* position in the heptad by a smaller Ala. The explanation is supported by a mild decrease in the thermal stability of the coiled-coil dimer containing Tpm residues 64–154 upon the Val95Ala substitution [36]. In contrast, the Ile92Thr substitution that also involves Tpm residue 92 in the *a* position in the heptade within the hydrophobic core of the coiled coil and significantly decreases its thermal stability is likely associated with familiar DCM [39]. Another HCM-associated mutation in this part of the Tpm molecule is Ala107Thr at the *b* position in the heptad [40].

There are also three HCM-associated mutations in the N-terminal part of the Tpm molecule, which are located, like Glu98Lys, at the *g* position in the heptad repeat (see Supplementary Figure S3). Among them, the Arg21His mutation is located in actin-binding pseudo-repeat 1, while the Ala63Val and Lys70Thr mutations are located in the pseudo-repeat 2 [4]. The Arg21His mutation was shown to significantly reduce the α-helical content of aTm1a1–28Zip, a chimeric peptide containing the first 28 residues of Tpm, and decrease the thermal stability of this peptide [41]. It was also shown that this mutation causes a twofold decrease in Tpm’s affinity for F-actin [42]. In this regard, the effects of the Arg21His mutation were quite different from those of the Glu98Lys mutation studied here, which did not influence the α-helical content of Tpm (Supplementary Figure S1) and Tpm’s affinity for F-actin (Figure 5A). As for the Ala63Val and Lys70Thr mutations, they decreased, although differently, the thermal stability of the N-terminal part of the Tpm molecule, as was shown by the CD measurements [43]. Their effect was different from that of the Glu98Lys mutation, which stabilized this part of the molecule (Figure 2). The Lys70Thr mutation (but not Ala63Val) decreased the Tpm affinity for F-actin [44]. On the other hand, both these mutations, Ala63Val and Lys70Thr, significantly increased the Ca^2+^ sensitivity of the sliding velocity of thin filaments in the in vitro motility assay while the substantial sliding velocity remained even at low Ca^2+^ concentrations (at *p*Ca > 7) [43]. These effects of HCM-associated mutations Ala63Val and Lys70Thr are similar to those observed for the Glu98Lys mutation (Figure 6A, Table 2).

### 3.4. Limitation of the Work

The E98K mutation described here was heterozygous (see Section 2.1). Presumably, the Tpm dimers with the Glu98Lys substitution in only one of two chains of the Tpm molecule were present in the cardiac muscle of the patient with HCM diagnosis, together with a mixture of the Tpm WT and the Tpm dimers with the substitution in both chains of the molecule. Previous studies showed that the properties of various Tpm dimers with a substitution in only one of two chains of the molecule can differ from those of the dimers, carrying substitutions in both chains [37,45,46]. In addition to the cross-dimer heterogeneity within a Tpm dimer, in the myocardium of heterozygous carriers of the Glu98Lys mutation, there should be heterogeneity among the different Tpm dimers along a Tpm strand on the surface of the actin filament. The possible effect of such heterogeneity is poorly understood. However, although our work was limited to studying various properties of the Tpm dimers with the Glu98Lys substitution in both chains and their comparison with those of the Tpm WT homodimers, such dimers presumably present in the patient’s myocardium in a concentration sufficient for causing the structural and function changes described here that result in restrictive left-ventricular filling in the patient with the Glu98Lys Tpm substitution.

## 4. Materials and Methods

### 4.1. Genetic Investigation

Genomic DNA samples were extracted from venous blood using Quick-DNA Miniprep Plus Kit (Zymo Research Corp., Irvine, CA, USA) according to the manufacturer’s instructions. Genetic study for the proband was performed using two custom-targeted gene panels with sets of oligoprimers designed automatically using Ion AmpliSeq Designer^®^ (Thermo Fisher Scientific, Waltham, MA, USA). Library preparation was performed using Ion AmpliSeq^®^ Library Kit 2.0 according to the manufacturer’s instructions (Thermo Fisher Scientific, Waltham, MA, USA). Sequencing of the 25 genes (*ACTC1, CRYAB, CTNNA3, DES, DSG2, DSP, DSC2, EMD, FLNC, JUP, LDB3, LMNA, MYBPC3, MYH7, MYL2, MYL3, PLN, PKP2, SCN5A, TAZ, TGFB3, TMEM43, TNNI3, TNNT2*, and *TPM1*) was performed using high-throughput semiconductor sequencing on an Ion PGM^TM^ System (Thermo Fisher Scientific, Waltham, MA, USA). The reads were preprocessed using Torrent Suite Software 5.6.0 and variant annotation web server Ion Reporter 5.12 (Thermo Fisher Scientific, Waltham, MA, USA). NGS sequencing reads were visualized using the Integrative Genomic Viewer (IGV) tool [47] with hg19 as a reference genome. All genetic findings detected by NGS in the proband were validated by capillary Sanger sequencing using Applied Biosystems^®^ 3500 Genetic Analyzer (Thermo Fisher Scientific, Waltham, MA, USA).

Pathogenicity evaluation for the identified genetic variants was carried out according to the guidelines of the American College of Medical Genetics (2015) [18]. Detection of the genetic variant in the *TPM1* gene in the proband and his clinically unaffected daughter was performed by capillary Sanger sequencing. Pathogenicity of the c.292G > A (p.E98K) variant was assessed following ACMG (2015) criteria [18].

### 4.2. Protein Preparations

The Tpm preparations used in this work were recombinant proteins, which have an Ala-Ser N-terminal extension to imitate naturally occurring N-terminal acetylation of Tpm [48]. Human E98K Tpm1.1 mutant was obtained in plasmid pMW172 by site-directed mutagenesis using Q5 DNA Polymerase (NEB, NewEngland BioLabs, Ipswich, MA, USA) with the following oligonucleotides: 5′-GAGGAAAAGTTGGATCGTGCCCAG-3′ as a forward primer (mutant codon is underlined) and 5′-AACCAGCTGGATGCGTCTGTTCAG-3′ as an adjacent primer. The resulting plasmid was verified by sequencing. Protein expression and purification were performed as described previously [21,49]. Tpm WT and Tpm E98K concentrations were determined spectrophotometrically at 280 nm using an E^1%^ of 2.7 cm^−1^.

Myosin was extracted from the left ventricle of the porcine heart by the standard method [50]. Actin was prepared from *m. psoas* of the rabbit by the established standard method [51]. After polymerization by the addition of 4 mM MgCl_2_ and 100 mM KCl to monomeric G-actin solution, filamentous actin (F-actin) was further stabilized by the addition of a 1.5-fold molar excess of phalloidin (Sigma Chemical Co., St. Louis, MO, USA). For experiments in the in vitro motility assay, F-actin was labeled with a 2-fold molar excess of TRITC-phalloidin (Sigma Chemical Co., St. Louis, MO, USA).

Recombinant human cardiac Tn complex composed of TnI, TnT, and TnC was provided by HyTest (Cat.# 8ITCR). The concentration of the Tn complex was determined spectrophotometrically at 280 nm using an extinction coefficient of 0.99 for the complex containing 1 mg/mL TnI.

### 4.3. Circular Dichroism (CD)

Far-UV CD spectra of Tpm species (1.0 mg/mL) were recorded at 5 °C on a Chirascan CD spectrometer (Applied Photophysics, Surrey, UK) in 0.02 cm cells. Measurements of thermal unfolding were performed as described earlier [12], by following the molar ellipticity of Tpm at 222 nm over a temperature range from 5 °C to 75 °C at a constant heating rate of 1 °C/min. All measurements were performed in 30 mM Hepes-Na buffer, pH 7.3, containing 100 mM NaCl and 2 mM DTT. The reversibility of the unfolding–refolding process was assessed by reheating the Tpm sample directly after it had been cooled from the previous temperature scan. The thermal unfolding of both Tpm species, WT Tpm and E98K Tpm mutant, was fully reversible.

### 4.4. Differential Scanning Calorimetry (DSC)

DSC experiments were performed as described [17] on a MicroCal VP-Capillary differential scanning calorimeter (Malvern Instruments, Northampton, MA 01060, USA) at a heating rate of 1 K/min in 30 mM Hepes-Na buffer, pH 7.3, containing 100 mM NaCl. The protein concentration was 2 mg/mL. The Tpm samples were reduced before DSC experiments by heating at 60 °C for 20 min in the presence of 3 mM DTT. After such a procedure, all Tpm samples were in an entirely reduced state [20]. The cross-linking of E98K Tpm was achieved by several subsequent heating–cooling procedures directly in the measuring cell of the calorimeter in the absence of reducing agents, as described earlier [22]. The thermal unfolding of Tpm species studied was fully reversible both in reduced and in cross-linked states, thus allowing deconvolution analysis of the heat sorption curves, i.e., their decomposition into separate thermal transitions (calorimetric domains). The deconvolution analysis was performed using Origin v. 7.5 software (MicroCal Inc., Northampton, MA, USA) by fitting the non-two-state model [19] to the data as described earlier [20].

### 4.5. Viscosimetry

The viscosity measurements were performed as described earlier [17] on a falling-ball micro-viscometer Anton Paar AMVn (Ashland, VA, USA) in a 0.5 mL capillary at 20 °C. The specific density of the Tpm solutions was measured with an Anton Paar DMA 4500 device (Ashland, VA, USA) and taken into account for accurate viscosity calculation. All measurements were performed at a Tpm concentration of 2 mg/mL in a 30 mM Hepes-Na buffer (pH 7.3) containing 100 mM NaCl and 4 mM DTT. The measurements for each Tpm sample were repeated three times, and the obtained values for Tpm viscosity over buffer viscosity were averaged.

### 4.6. Cosedimentation of Tpm Species with F-Actin

The apparent affinity of Tpm species to F-actin was estimated using a co-sedimentation assay (see Supplementary Figure S2) as described earlier [12,17,21]. The solutions contained 10 µM F-actin and Tpm in concentrations increasing from 0.5 to 7.5 µM in 30 mM Hepes-Na buffer (pH 7.3) with 200 mM NaCl. The mixture was incubated 40 min at 25 °C and then subjected to ultracentrifugation for 40 min at 100,000 g at 4 °C. Supernatants and pellets were analyzed by SDS-PAGE (see Supplementary Figure S2). Five measurements were performed for each Tpm sample.

### 4.7. Temperature Dependences of Light Scattering

Thermally induced dissociation of Tpm complexes with F-actin stabilized by phalloidin was detected by changes in light scattering at 90°, as described earlier [12,17,21]. The solutions contained 20 µM F-actin and 10.5 µM Tpm in 30 mM Hepes-Na buffer (pH 7.3) with 100 mM NaCl. The experiments were performed at a wavelength of 350 nm on a Cary Eclipse fluorescence spectrophotometer (Varian Australia Pty Ltd., Mulgrave, VIC, Australia) equipped with a temperature controller and thermoprobes. All measurements were performed at a constant heating rate of 1 °C/min. The dissociation curves were fitted by a Boltzmann sigmoidal decay function. The main parameter extracted from this analysis is *T_diss_*, i.e., the temperature at which a 50% decrease in light scattering occurs.

### 4.8. In Vitro Motility Assay

The effects of the E98K Tpm substitution on the calcium regulation of actin-myosin interaction were studied in the in vitro motility assay as described earlier [17]. In brief, 300 µg/mL myosin in AB buffer (25 mM KCl, 25 mM imidazole, 4 mM MgCl_2_, 1 mM EGTA, and 20 mM DTT, pH 7.5) containing 0.5 M KCl was loaded into the experimental flow cell. After 2 min, 0.5 μg/mL BSA was added for 1 min. Further 50 µg/mL of non-labeled F-actin in AB buffer with 2 mM ATP was added for 5 min to block nonfunctional myosin heads. To form regulated thin filaments, 10 nM TRITC-phalloidin labeled F-actin and 100 nM of Tpm and Tn were added for 5 min. Finally, the cell was washed with AB buffer, containing 0.5 mg/mL BSA, oxygen scavenger system, 20 mM DTT, 2 mM ATP, 0.5% methylcellulose, 100 nM Tpm/Tn, and appropriate Ca^2+^/EGTA in proportions calculated with the Maxchelator program. The experiments were done at 30 °C. The sliding velocity was analyzed with GMimPro software [52].

To investigate the effect of the E98K Tpm substitution on the cooperativity of the myosin interaction with regulated thin filaments, the dependence of the sliding velocity of the filament on the myosin concentration *c* was analyzed as described [26]. The surface density of myosin was varied by the infusion of different myosin concentrations in the flow cell. The experiments were repeated 3 times, and the movement of 50–100 filaments was measured in each experiment at each myosin concentration. The dependence of the sliding velocity *V* of the regulated thin filament on the concentration of myosin added to the flow cell was fitted with the Hill equation [53]:

*V* = *V_max_* × *c^h^* × (*c*_50_*^h^* + *c^h^*)^−1^ where *V_max_* is maximal sliding velocity, *c* is myosin concentration, *c*_50_ is the concentration required to achieve half-maximal velocity, and *h* is the Hill cooperativity coefficient.

### 4.9. Molecular Dynamics (MD) Simulation and Analysis

The MD simulations were performed using GROMACS v. 2019.3 or 2021.5 [54]. The model system was placed in a rectangular box extending at least 15 Å in each direction from the protein. The box was filled with water molecules using the TIP3P water model, and Na^+^ and Cl^−^ ions were added to ensure a net zero charge and ionic strength of 0.15 M. The energy minimization, the NVT and NPT equilibrations, and the MD simulation were carried out using the AMBER99SB-ILDN force field [55] for Tpm alone or CHARMM36 force field [56] for the F-actin–Tpm–Tn complex. The temperature was set to 300 K. The duration of the MD runs was 204.8 ns with a step of 2 fs, and the frames (snapshots of the MD trajectory) were recorded every 200 ps.

The structure of a full α-Tpm molecule (Sus scrofa, PDB code 1C1G) [57] was used as a starting Tpm model. Several residue substitutions were made with the UCSF CHIMERA package [58,59] to build a human WT Tpm1.1 (α-Tpm) model. The occupancy of the backbone hydrogen bonds (h-bonds) was calculated with a Python script and the h-bond function of GROMACS. The occupancy values for identical residues of both Tpm chains were averaged, then time-averaged for the second half of the MD trajectory and plotted against the residue number to characterize the α-helix stability along the molecule. The terminal residues were excluded from the analysis as they were subjected to a larger disordering in the MD simulation.

The simulation of the F-actin–Tpm–Tn complex was described in detail in [27]. Refined model 1, used in this study, was constructed from the 6kn7 structure of F-actin with Tpm and Tn in the blocked state [28] combined with the refined Tpm and TnI structural data [60]. The model contains four Tpm dimeric molecules (two from each of the Tpm-Tn strands on both sides of F-actin) with the overlap junctions between the N- and C-termini of adjacent Tpm-molecules and all the components of the Tn complex except the N-terminal residues 1–86 of TnT not resolved in any published cryo-EM electron density maps. TnT residues 151–199, also not resolved in the cryo-EM structures, were reconstructed with a structure prediction software and included in the MD simulation.

## 5. Conclusions

We identified a novel genetic variant c.292G > A (p.E98K) in the *TPM1* gene encoding cardiac Tpm that was recently registered in the ClinVar database and assigned to HCM without a description of clinical data and/or effects of the mutation on the structural and functional properties of Tpm. Our detailed clinical investigations revealed that this mutation leads to HCM with a pronounced restrictive phenotype accompanied by diastolic dysfunction and slow progressive neuromuscular involvement. To understand the molecular mechanism by which this mutation impairs cardiac function, we produced recombinant Tpm carrying an E98K substitution and applied various methods, such as CD, DSC, in vitro motility assay, etc., to investigate how this substitution affects the structure of the Tpm molecule and its functional properties. The DSC results showed that the E98K substitution in the N-terminal part of the Tpm molecule significantly destabilizes the C-terminal part of the molecule, thus indicating a long-distance destabilizing effect of the substitution on the Tpm coiled-coil structure (Figure 3, Table 1), and this effect was confirmed by molecular dynamics (MD) simulation (Figure 4). The in vitro motility assay studies showed that the E98K substitution significantly impaired Tpm’s regulatory properties by increasing the Ca^2+^ sensitivity of the sliding velocity of regulated thin filaments over cardiac myosin. Moreover, it caused an incomplete block of the thin filament sliding at low Ca^2+^ concentrations (Figure 6A, Table 2). The latter can be explained by the MD simulation (Figure 7) showing that the E98K substitution would loosen the Tpm interaction with the inhibitory domain of TnI, thus increasing Tpm mobility on the surface of the actin filament and leading to a partial unlocking of the myosin binding sites. The results allowed us to explain, at least partly, those molecular mechanisms by which the E98K mutation in the *TPM1* gene impairs myocardial relaxation and leads to such a severe human cardiac disease as complex cardiomyopathy.

## Figures and Tables

**Table 1:** Calorimetric parameters obtained from the DSC data for individual thermal transitions (calorimetric domains) of WT Tpm and Tpm with mutation E98K ^a^.

Tpm	*T_m_* ^b^ (°C)	Δ*H*_cal_ (kJ mol^−1^)	Δ*H*_cal_ (% of Total)	Total Δ*H*_cal_^c^ (kJ mol^−1^)
Tpm WT Reduced				1330
Domain 1	38.2	350	26	
Domain 2	43.3	480	36	
Domain 3	50.7	500	38	
Tpm E98K Reduced				950
Domain 1	30.9	65	7	
Domain 2	39.0	390	41	
Domain 3	52.9	495	52	
Tpm E98K Cross-linked				865
Domain 1	30.6	65	7	
Domain 3	51.6	645	75	
Domain 4	57.9	155	18	

^a^ The parameters were extracted from the DSC curves shown in Figure 3. ^b^ The error of the given values of transition temperature (*T_m_*) did not exceed ±0.2 °C. ^c^ The relative error of the given values of calorimetric enthalpy, Δ*H*_cal_, did not exceed ± 10%.

**Table 2:** Characteristics of the *p*Ca-velocity relationship of the thin filament containing WT Tpm or E98K Tpm in the in vitro motility assay.

Tpm	*V_*max*_* (µm/s)	*V_*0*_* (µm/s)	*p*Ca_50_	*n*
WT Tpm	1.85 ± 0.01	0	5.85 ± 0.01	1.7 ± 0.2
E98K Tpm	1.37 ± 0.02 *	0.44 ± 0.04 *	6.00 ± 0.05 *	1.3 ± 0.1 *

*V*_max_, the maximum sliding velocity of thin filaments at saturating Ca^2+^ concentration; *V*_0_, the sliding velocity at *p*Ca 7.5; *p*Ca_50_ is the *p*Ca value at which the sliding velocity is half-maximal; *n*, Hill cooperativity coefficient. The symbol (*) denotes statistically significant differences in characteristics of the filaments containing E98K Tpm from those with WT Tpm, *p* < 0.05 (*t*-test).

## Data Availability

The raw clinical data supporting this article cannot be placed in a public repository due to ethical reasons (personal data protection) but it will be available by co-author (E.V.Z., zhelene@mail.ru) upon reasonable request. The genetic data is under registration in the public repository ClinVar https://www.ncbi.nlm.nih.gov/clinvar/variation/1472862/ (accessed on 22 July 2023)). Accession numbers for this variant will be provided upon registration. All experimental data generated or analyzed during this study are included in this article.

## Supplementary Materials

The following supporting information can be downloaded at: https://www.mdpi.com/article/10.3390/ijms241512359/s1.
