# Supplementary material for: Novel Mutation Glu98Lys in Cardiac Tropomyosin Alters Its Structure and Impairs Myocardial Relaxation
Source: Int J Mol Sci. 2023 Aug 2;24(15):12359. doi: 10.3390/ijms241512359 (PMC10419091; doi:10.3390/ijms241512359)
Supplement: Supplementary file 1 [file ijms-24-12359-s001.zip › ijms-2511373-supplementary.pdf]

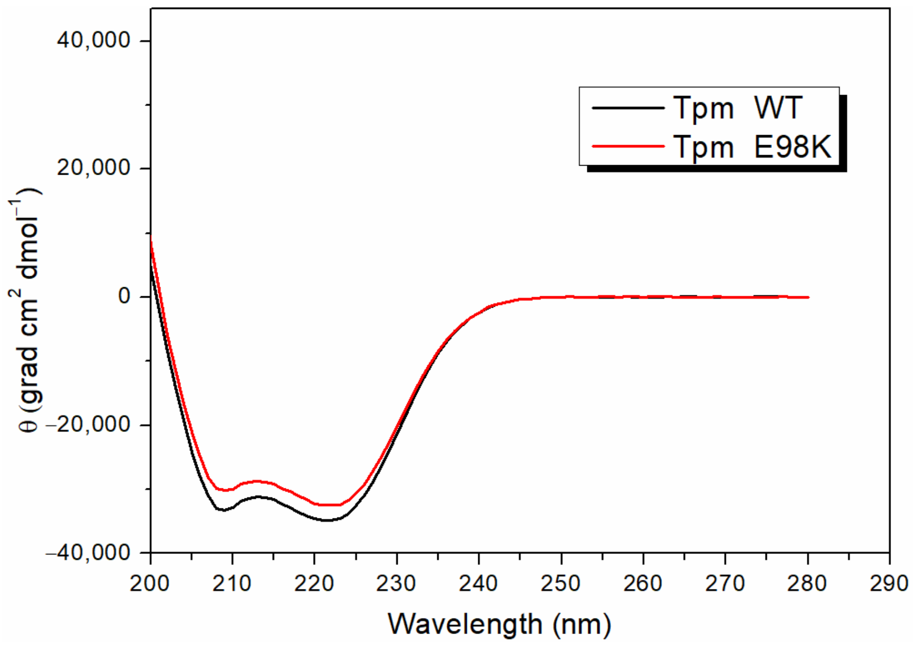

Figure S1. CD spectra of the Tpm E98K compared with Tpm WT. Spectra were recorded at 10 °C.

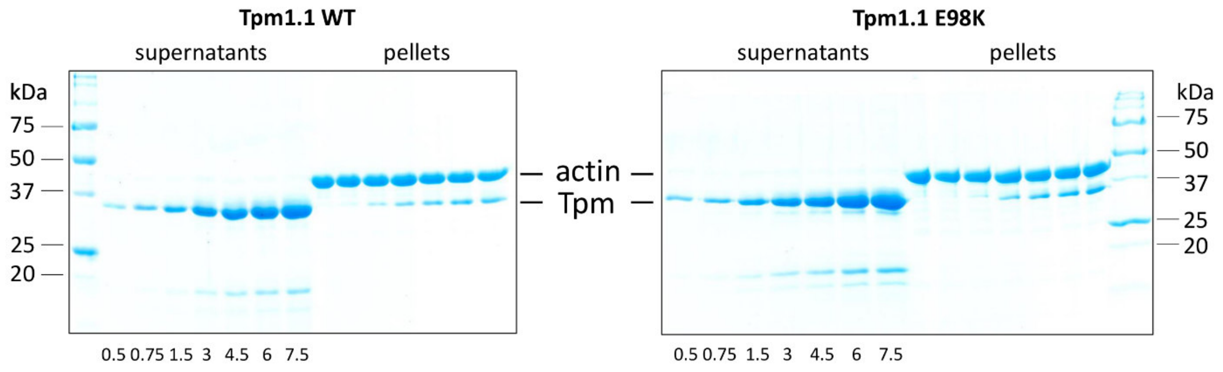

Figure S2. Representative SDS-PAAG gels showing supernatants and pellets obtained from co-sedimentation of Tpm WT and E98K Tpm with F-actin. Total concentrations (µM) of Tpm in the samples are listed under the gels. It is clear that the E98K substitution does not significantly affect the Tpm affinity to F-actin.

abc***d***efg

1 **M**DA**I**KKK
MQM**L**KLD
KEN**A**LD**R** 21
AEQ**A**EAD
KKA**A**EDR
SKQ**L**EDE
LVS**L**QKK
LKG**T**EDE
LDK**Y**SE**A** 63
LKD**A**QE**K** 70
LEL**A**EKK
ATD**A**EAD
VAS**L**NRR
IQL**V**EE**E** 98
LAT**A**LQK
LEE**A**EKA
ADE**S**ERG
MKV**I**ESR
AQK**D**EEK
MEI**Q**EIQ
LKE**A**KHI
AED**A**DRK
YEE**V**ARK
LVI**I**ESD
LER**A**EER
AEL**S**EGK
190 **C**AE**L**EEE
LKT**V**TNN
LKS**L**EAQ
AEK**Y**SQK
EDR**Y**EEE
IKV**L**SDK
LKE**A**ETR
AEF**A**ERS
VTK**L**EKS
IDD**L**EDE
LYA**Q**KLK
YKA**I**SEE
LDH**A**LND
MTS**I** 284

Figure S3. Heptad repeats (*abcdefg*)$_n$ in the amino acid sequence of cardiac Tpm1.1 isoform. Hydrophobic residues at positions *a* and *d* that form a hydrophobic core of the molecule are shown in bold.
The residues R21, A63, K70, and E98 at *g* position in the N-terminal part of Tpm, whose substitutions with His, Val, Thr, and Lys residues, respectively, by HCM-associated mutations R21H, A63V, K70T, and E98K in the *TPM1* gene are shown in red. The only Cys residue of Tpm1.1 (C190) is shown in blue.
